# Fecal Carriage and Epidemiology of Extended-Spectrum Beta-Lactamase/Carbapenemases Producing Enterobacterales Isolates in Bulgarian Hospitals

**DOI:** 10.3390/antibiotics10060747

**Published:** 2021-06-20

**Authors:** Rumyana Markovska, Petya Stankova, Temenuga Stoeva, Dobrinka Ivanova, Daniela Pencheva, Radka Kaneva, Lyudmila Boyanova

**Affiliations:** 1 Department of Medical Microbiology, Medical Faculty, Medical University of Sofia, Georgi Sofiiski, 1431 Sofia, Bulgaria; petya.stankova88@abv.bg (P.S.); l.boyanova@hotmail.com (L.B.); 2 Department of Microbiology and Virology, Medical University of Varna, St. Marina, 9002 Varna, Bulgaria; temenuga.stoeva@abv.bg; 3 Second Multiprofile Hospital for Active Treatment, 1431 Sofia, Bulgaria; micro_vtora.mbal@abv.bg; 4 Molecular Medicine Center, Medical University of Sofia, 1431 Sofia, Bulgaria; dacheva@mmcbg.org (D.P.); kaneva64@yahoo.com (R.K.)

**Keywords:** fecal carriage, ESBL, carbapenemases, Enterobacterales

## Abstract

The gastrointestinal tract is an important reservoir of extended spectrum beta-lactamase (ESBL)/carbapenemase-producing Enterobacterales isolates. This study included patients from two Bulgarian hospitals. Overall, 98 ESBL producers (including 68 *Escherichia coli* and 20 *Klebsiella pneumoniae* isolates) were detected among 99 hospitalized patients, 212 patients at admission, and 92 hospital staff in 42.4%, 24.5%, and 4%, respectively. We observed *bla*_CTX-M-15_ in 47% of isolates, *bla*_CTX-M-3_ in 39% and *bla*_CTX-M-14_ in 11%. Three *bla*_CTX-M-15_ positive isolates were also *bla*_KPC-2_ positive. High transferability was detected for *bla*_CTX-M-3_ carrying plasmids (55%) with L/M and I1 replicon plasmids, followed by CTX-M-14 (36.4%) and CTX-M-15 (27.9%) with IncF plasmids. BlaKPC-2 was carried by FIIAs plasmids. Epidemiology typing revealed 8 *K. pneumoniae* ST types—ST15(8/20), ST17(4/20), ST37(2/20) and 9 *E. coli* ST types—ST131 (30.9%, 21/68), ST38 (8/68), ST95(7/68) and ST316(7/68). All ST131 isolates but one was from the highly virulent epidemic clone O25bST131. This is the first report in Bulgaria about ESBL/carbapenemase faecal carriage. We observed high ESBL/carbapenemases prevalence. A predominant number of isolates were members of highly epidemic and virulent PanEuropean clones ST15 *K. pneumoniae* and O25bST131 *E. coli*. High antibiotics usage during the COVID pandemic will worsen the situation. Routine screenings and strict infection control measures should be widely implemented.

## 1. Introduction

Members of order Enterobacterales are Gram-negative bacteria normally inhabiting the intestinal tract. These bacteria are also the most common cause of nosocomial and community acquired infections [1,2,3]. The increasing rates of antibiotic resistance among them, especially in *Escherichia coli*, *Klebsiella pneumoniae*, and *Enterobacter cloacae* complex, have been globally reported [2,4,5]. The resistance was mostly associated with production of beta-lactamases (extended-spectrum beta-lactamases (ESBL) or/and carbapenemases) [2]. ESBLs could be classified into three main groups, TEM, SHV and CTX-M, with CTX-M-15 and CTX-M-14 being the most widespread variants [2]. Extensive usage of carbapenems for treatment of infections caused by ESBL producers has led to a sharp increase of carbapenemase producers (such as KPC, NDM, and VIM) [6]. Different mobile elements (plasmids and integrons) contribute to the dissemination of the producers. In the mobile elements, ESBL and carbapenemases genetic determinants are located with other resistance genes causing nonsusceptibility to beta-lactams and many other groups such as quinolones, aminoglycosides, tetracyclines, and trimethoprim/sulfamethoxazole [7].

The gastrointestinal tract is an important reservoir of ESBL/carbapenemase-producing enterobacteria [8]. It is also a hot spot where can occur exchanges of resistance genes and antibiotic pressure could result in appearance of resistant mutants [8]. This can lead to life-threatening nosocomial and community-acquired infections [8,9]. Fecal carriage of ESBL producers was widely reported all over the world [8,9]. In next decades such reports increased [8]. Wide differences in ESBL/carbapenemase fecal carriage rates have been reported in different countries, suggesting dynamics of their geographical evolution [8,10,11]. In Bulgaria, fecal carriage of ESBL/carbapenemase-producing Enterobacterales has not been investigated so far.

The aim of this study was to investigate the prevalence of ESBL and carbapenemase-producing Enterobacterales from fecal samples, collected from hospitalized patients (>48 h hospital-stay), and medical personnel in Varna hospital and from patients at admission in Sofia hospital, to discover the clonal relatedness between isolates and distribution of MLST types among *E. coli* (Achtman scheme) and *K. pneumoniae* (Pasteur scheme) isolates, as well as to characterize mechanisms of beta-lactam resistance.

## 2. Material and Methods

### 2.1. Bacterial Isolates

The study was conducted in two hospitals—University Hospital “Saint Marina”, Varna and Second City hospital in Sofia during the period January 2015–September 2015 (January–March 2015 for UH Varna and May-September 2015 for Sofia hospital). In University hospital in Varna, routine screening of 99 hospitalized patients in ICU words (>48 h after admission) was performed and in Sofia, 212 patients at the day of their admission were investigated. The fecal samples were sequentially collected, one per patient. They were inoculated on selective MacConkey agar with cefotaxime 1 mg/L and on Chromagar^TM^KPC (Becton Dickinson, Springfield, IL, USA). Bacterial isolates were identified using routine biochemical identification and were confirmed by VITEK (bioMérieux, Marcy L’Étoile, France)) or Phoenix (Becton Dickinson, Springfield, IL, USA). Antimicrobial susceptibility testing was performed using the disk diffusion method on Müller-Hinton II agar and the interpretation was by clinical breakpoints of the European Committee on Antimicrobial Susceptibility Testing (EUCAST) guidelines (Version 10) (http://www.eucast.org/clinical_breakpoints/ accessed on 21 April 2021). The following antibiotics were tested: amoxicillin-clavulanic acid, piperacillin-tazobactam, cefotaxime, ceftazidime, cefepime, cefoxitin, imipenem, tobramycin, gentamicin, amikacin, trimethoprim/sulfamethoxazole, ciprofloxacin, and levofloxacin.

### 2.2. Phenotypic ESBL and Carbapenemases Detection

Presumptive ESBL production was detected with the double-disk synergy method [12]. In the case of nonsusceptibility to carbapenems or/and growth on selective Chromagar^TM^KPC media, a phenotypic confirmation of carbapenemase production was performed by the modified Hodge test and by the KPC/Metallo-beta-lactamase and OXA-48 Confirm Kit (ROSCO Diagnostica, Taastrup, Denmark). The beta-lactamase production was analyzed by isoelectric focusing (IEF) and the hydrolytic activity of individual beta-lactamase bands was assessed by a bioassay as previously described [13].

### 2.3. Molecular-Genetic Beta-Lactamase Identification

All isolates were screened for the presence of *bla*_VIM_, *bla*_IMP_, *bla*_KPC_, *bla*_NDM_, *bla*_OXA-48,_ *bla*_CTX-M_, and *bla*_SHV_, as previously described [12,13,14,15]. The genes were sequenced using primers binding outside the coding region of *bla*_SHV_, *bla*_CTX-M-1_-group, *bla*_KPC_ [12]. The nucleotide and deduced amino acid sequences were analyzed and multiple alignments were performed using Chromas Lite 2.01 (Technelysium Pty Ltd., Brisbane, Australia) and DNAMAN version 8.0 Software (Lynnon BioSoft, Vaudreuil-Dorion, QC, Canada).

### 2.4. Conjugation Experiments and Replicon Typing

Conjugation experiments were carried out as previously described [12]. Plasmid replicon typing were performed according to the Carattoli scheme [16]. *E. coli* phylotyping was done by Clermont scheme [17].

### 2.5. ERIC and MLST Typing

The clonal relatedness was investigated by ERIC PCR and Multilocus Sequence Typing (MLST). ERIC-1 and -2a primers were used [13]. Primers and protocols for *E. coli* MLST, Achtman scheme, were reported by Wirth et al. [18]. The assignment to allelic numbers and sequence types (STs) were performed according to the MLST database (https://bigsdb.web.pasteur.fr/ecoli/ecoli.html, accessed on 10 January 2021). Additionally, the *pabB* gene was detected to prove the presence of O25bST131 clone according to the Clermont scheme [19].

For the *K. pneumoniae* isolates, protocols and assignment to allelic numbers and sequence types (STs) were carried out as described in the MLST database (Pasteur Institute, Paris, France; http://bigsdb.web.pasteur.fr/klebsiella/klebsiella.html accessed on 10 January 2021). A clonal complex was defined as a group of two or more independent isolates that shared six identical alleles.

## 3. Results

### 3.1. Bacterial Isolates

A total of 100 Enterobacterales isolates resistant to cefotaxime were isolated from the fecal samples (one per patient) of 99 patients, hospitalized in UH Varna (from ICU wards) and from the 212 patients, at the day of admission, in Second Town hospital (non-ICU wards)—Sofia during their admission. Ninety-two members of all medical personnel in ICU wards in “Saint Marina” hospital, Varna were studied. The obtained isolates were identified as *Escherichia coli*, *n* = 68; *Klebsiella pneumoniae*, *n* = 20; *Enterobacter cloacae*, *n* = 6; *Klebsiella aerogenes (formerly Enterobacter aerogenes)*, *n* = 3, *Shigella sonnei*, *n* = 2, and *Citrobacter freundii*, *n* = 1.

### 3.2. Antimicrobial Susceptibility Testing

**Figure 1:**
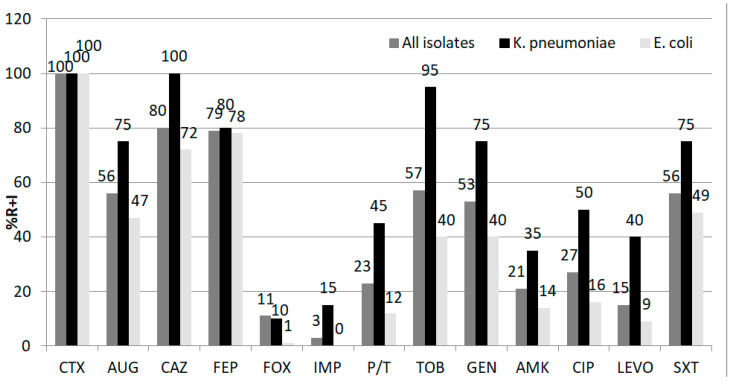
Antimicrobial susceptibility rates in the whole group of Enterobacterales isolates (*n* = 98), *E. coli* (*n* = 68), and *K. pneumoniae* (*n* = 20). Abbreviations: CTX, cefotaxime; AUG, amoxicillin/clavulanic acid; CAZ, ceftazidime; FEP, cefepime; FOX, cefoxitine; IMP, imipenem; P/T, piperacillin/tazobactam; TOB, tobramycin, GEN, gentamycin; AMI, amikacin; CIP, ciprofloxacin; LEVO, levofloxacin; SXT, trimethoprim/sulfamethoxazole; R + I—resistant and intermediately susceptible isolates.

The nonsusceptibility rates (resistant and intermediately susceptible) of the whole group of isolates and separately for *E. coli* and *K. pneumoniae* are shown in Figure 1.

### 3.3. Phenotypic ESBL and Carbapenemases Detection

The ESBLs production was confirmed with double disk synergy test for 95 isolates, two *E. cloacae* isolates were possible AmpC hyperproducers (FOX-reistant and with antagonism shown when disk amoxicillin/clavulanic acid was placed near cefotaxime or ceftazidime). Three isolates resistant to imipenem gave positive modified Hodge test and increasing zone with disk meropenem+boronic acid suggesting class A carbapenemases activity.

### 3.4. Molecular-Genetic Beta-Lactamase Identification

The PCR and sequencing revealed the presence of *bla*_ESBL_ in 98 isolates and genes, encoding carbapenemases in 3 of them. The prevalence of (solely) ESBL-producing Enterobacterales was 30.2% (94 from 311) among patients (hospitalized and at admission) and 4% (4 from 92) among hospital staff. Three isolates from hospitalized patients (1%) produced carbapenemases as well. Among the hospitalized patients, the ESBL producer rate was 42.4% (42 of 99 patients) and among the patients at admission, they were 24.5% (52 of 212 patients). The difference was statistically significant (*p* = 0.0014).

We observed *bla*_CTX-M-15_ in 47% (46 from 98) of isolates and *bla*_CTX-M-3_ in 39% (38 from 98) (Table 1). Three *bla*_CTX-M-15_ positive isolates were also positive for *bla*_KPC-2_. According to the species, 41% (28/68) of *E. coli* and 70% (14/20) of *K. pneumoniae* were positive for *bla*_CTX-M-15_ (including KPC-2 producers); for *bla*_CTX-M-3_—40% of *E. coli* (27/68) and 30% of *K. pneumoniae* (6/20) were positive. Eleven (11%) isolates, all of them *E. coli*, showed the presence of *bla*_CTX-M-14,_ two of *bla*_SHV-12_ and one of *bla*_CTX-M-1_.

### 3.5. Isoelectric Focusing and Bioassay

The isoelectric focusing was carried out in representative isolates for the species and the center and confirmed the beta-lactamase production of the ESBLs and carbapenemases. Of 20 *K. pneumoniae* isolates, 10 were subjected to IEF (three of the CTX-M-3-producing, four of the CTX-M-15-producing and all the KPC-2-producing isolates). We detected single band hydrolyzing cefotaxime with pI8.8 in *bla*_CTX-M-15_ positive, with pI8.4 in *bla*_CTX-M-3_ positive. For *bla*KPC-2 positive isolates two bands, hydrolyzing cefotaxime, were observed—with pI8.8 and with pI6,7. The band with pI 6.7 additionally hydrolyzed imipenem. Ten of the 27 *bla*_CTX-M-3_ *E. coli* isolates 11 from 22 *bla*_CTX-M-15_ positive and 6 from 11 *bla*_CTX-M-14_ positive isolates gave cefotaxime hydrolyzing band at pI8.8, pI8.4 and pI8.0, respectively. One *E. coli* isolate showed cefotaxime hydrolyzing band at pI8.2 (SHV-12) and another one at pI8.4 (CTX-M-1). All isolates have only one cefotaxime hydrolyzing band. There were not isolates with two ESBLs. *Enterobacter* isolates also produced only one cefotaxime hydrolyzing band.

### 3.6. Conjugation Experiments and Replicon Typing

Conjugation experiments showed 41 transconjugants. The most transferable gene was *bla*_CTX-M-3_ in 55% (23 of 38 donors), followed by *bla*_CTX-M-14_ in 36.4% (4 from 11) and *bla*_CTX-M-15_ in 27.9% (12 from 43 donors). We detected transfer of *bla*_KPC-2_ in two of three KPC-2-producing donors. The susceptibility phenotypes and replicon types of the transconjugants are shown in Table 2 and Table 3. *bla*_CTX-M-3_ was mainly associated with I1 (*n* = 11) and L/M (*n* = 10) replicons. Only single isolates showed FIIAs. *bla*_CTX-M-15_ was carried by IncF type (*n* = 8), HI2 (*n* = 1) and non-typeable plasmids (*n* = 3). FIIAs replicons were specific for *bla*_KPC-2_ carrying plasmids. Two isolates (1 CTX-M-3-producing *E. cloacae* complex and 1 *K. aerogenes*) gave transconjugants with L/M replicon type and CTX, TOB, GEN, AMI, SXT resistotype. The single *C. freundii* isolate gave CTX, TOB. GEN, CIP, TET, SXT transconjugant with HI2 replicon type.

### 3.7. MLST and ERIC Typing

Epidemiology typing revealed 8 ERIC clusters in *K. pneumoniae* isolates, they had between 1 and 7 members, which corresponded to 8 ST types—ST15(8 isolates, 40%), ST17(4 isolates), ST37(2 isolates), ST873(2 isolates) and ST215, ST359, ST340, ST16 as single isolates (Table 2). ST15 *K. pneumoniae* isolates were prevalent among hospitalized patients and were associated with CTX-M-15 and KPC-2 production (Table 1 and Table 2). In this clone, two *K. pneumoniae* CTX-M-15-producing isolates from the medical staff were also included.

Among *E. coli* isolates, 20 ERIC types were detected, nine of them had between 2 and 21 members. They corresponded to 9 main ST types (Table 3)—ST131*_n_*_=21 (type f)_ (30.9%), ST38*_n_*_=8 (type b)_, ST95*_n_*_=7 (type a)_, ST316*_n_*_=7 (type l)_, ST43*_n_*_=2 (type c)_, ST540*_n_*_=2 (type n)_, ST676*_n_*_=5 (type m)_, ST405 *_n_*_=2 (type d)_, ST517*_n_*_=3 (type t)_. Eleven isolates represented unique ERIC types and MLST have not been performed. All ST131 *E. coli* isolates but one were positive for *pabB* gene.

ST131 *E. coli* (*n* = 21) was detected predominantly among patients at admission and was less prevalent among hospitalized patients, and was associated with production of CTX-M-15 (38.1%, 8/21), CTX-M-3 (47.6%, 10/21) and CTX-M-1, CTX-M-14 and SHV-12 as single isolates.

The following *E. coli* phylotypes were identified: B2*_n_*_=33_ (48.5%, 33/68), A*_n_*_=21_, B1*_n_*_=4_, D*_n_*_=10_. The association between MLST types and phylotypes is shown in Table 3. The B2 group included members of ST131, ST95, ST 676, D group—members of ST38 and ST405, A group—ST316, ST43, almost all the unique ERIC types and B1—ST517.

With exception of two CTX-M-15-producing *E. cloacae* isolates, all demonstrated unique ERIC types *S. sonnei* generated only three bands in ERIC PCR, so, they were not enough to compare the isolates. 

## 4. Discussion

To the best of our knowledge, this is the first report on intestinal carriers of ESBL/carbapenemase-producing Enterobacteriales isolates in Bulgaria. This study revealed a high (30.2%) rate of fecal ESBL producers (including 3% carbapenemase producers) among 311 Bulgarian patients. Importantly, the rate of ESBL-producing strains among hospitalized patients (99 patients) was statistically higher compared with that in patients at admission (212 patients) (42.4% vs. 25.5%). The ESBL prevalence among patients at admission could reflect the frequency of ESBL producers in the community and it was higher than that in other European countries [10]. Our results were higher than those in Portuguese hospitals, where authors compared ESBL frequency among hospitalized, >48 h hospital stay (24%) and patients at admission (17%) [20]. Similar results have been reported in French hospitals—with 17.7% ESBL gut carriage [21]. Our rates were much higher than those published from other European countries (for patients at admission) such as Belgium—11.6% [22], UK—9.5% [23], Spain—7.7% [24] and The Netherlands—8.2% [25]. All these data were in concordance with a very high level of cephalosporin 3rd resistant invasive isolates in Bulgaria. In 2019 ECDC reports the highest frequency of resistance to cephalosporin third generation in invasive isolates of *E. coli* (38.6%), and *K. pneumoniae* from Bulgaria (75.7%). Unfortunately, the frequency of these isolates in Bulgaria was the highest in Europe and has remained high over the last five years (https://www.ecdc.europa.eu/sites/default/files/documents/Additional-tables-EUEEA-population-weighted-mean-2019.pdf, accessed on 23 April 2021). Many reports focused on increasing rates of gut colonization with ESBL/carbapenemase producers among patients infected by ESBL/carbapenemase-producing microorganisms [26]. The high frequency of ESBL intestinal carriage is a threat of an increase in infections caused by ESBL producers. The rate of ESBL gut carriers in our study was much lower among the medical personnel (4%), but their appearance showed that medical staff can also contribute to the dissemination of these isolates.

A reason for the high frequency of fecal ESBL-producing Enterobacteriales isolates in the present study and cephalosporin third generation resistant invasive isolates in Bulgaria could be a high level of cephalosporin and carbapenems consumption in our country. Bulgaria was the first place of usage of third generation cephalosporins in Europe with 57% of the total antimicrobial consumption and in last place for penicillin usage (7%) in 2019 (https://www.ecdc.europa.eu/sites/default/files/documents/Antimicrobial-consumption-in-the-EU-Annual-Epidemiological-Report-2019.pdf, accessed on 23 April 2021). Strict measures for infection control and good compliance with antibiotic policy are necessary.

Our isolates had the following resistance rates to non—beta-lactam antimicrobials: 21–57% for aminoglycosides and 15–27% for quinolones. The resistance rates for these agents were much higher in *Klebsiella* than in *E. coli* isolates (Figure 1). Only three isolates from the studied group (*K. pneumoniae*) were carbapenem-resistant. Carbapenems, amikacin, and levofloxacin are adequate options for empirical antimicrobial therapy when necessary. Piperacillin/tazobactam can also be considered after antimicrobial susceptibility testing. Monitoring of the patients colonized by ESBL/carbapenemases producers should be regularly performed.

As in other reports [8,10], we detected the prevalence of *E. coli* ESBL-producing fecal isolates (69%), and in less proportion *Klebsiella* ESBL-producing isolates (20.4%). Similar to other studies we found a predominance of CTX-M-1 group producers [8,10]. The CTX-M-15 in the present study was predominantly found among *K. pneumoniae* and *E. cloacae* complex isolates (Table 1). *E. coli* isolates produced CTX-M-15 and CTX-M-3 in almost equal proportions, in 41% and 40%, respectively. We could assume that *bla*_CTX-M-15_ allele might offer the bacteria a selective advantage to overcome antibiotic pressure in hospitals. CTX-M-14 enzyme has been previously detected in Bulgaria only sporadically [27]. Interestingly, in this study, we observed it among *E. coli* and in only 16%. Gut colonization with CTX-M-15 producers has been reported elsewhere [8,10] (including gut carriers), whereas CTX-M-9 and CTX-M-14 from the same origin have been reported in few countries—Spain and China [8].

Only three isolates *K. pneumoniae* (3%) produced KPC-2 carbapenemases and they were in combination with CTX-M-15 production. In Europe, the rates of carbapenemase producers has increased during the last years [5,6] and one important contributor for this is intestinal carriage of carbapenemases producers. The presence of carbapenemase carriers is of high importance since carbapenemase producers could not be easily cleared from the body. The process can take several months (>387 days) [28].

In the present study, ST15 was the main MLST type among *K. pneumoniae* and was associated with CTX-M-15 and KPC-2 production. The ST15 clone is an interesting PanEuropean clone reported to produce wide range of ESBL and carbapenemases [29,30,31,32,33]. Among the hospitalized patients, this clone could be an important driver of KPC-2 production and/or CTX-M-15 production. In the present study, 8 of 20 investigated *K pneumoniae* isolates were from this clone and three of them produced KPC-2. It is also very important that two isolates from the medical staff had CTX-M-15-producing ST15 *K. pneumoniae*. In Bulgaria, during the period 2012–2015, clinical KPC-2-producing ST15 *K. pneumoniae* isolates were detected in Varna hospital [13]. Our data shows the great transmissible potential of ST15 isolates including those from gut colonization. This comes to show that the gut microflora can act as a reservoir of carbapenemase producers, therefore, screening for fecal carriage is important, especially for immunosuppressed and transplanted patients.

The other intestinal ST *K. pneumoniae* types in this study were ST17 and ST37. They produced CTX-M-3 enzymes among hospitalized patients. These STs have also been isolated previously from clinical isolates in Bulgaria [34], but were mostly associated with CTX-M-15 production. We could suggest that additionally to clonal propagation, plasmid distribution plays an important role for ESBL determinants dissemination. Similar to other reports in our study *bla*_CTX-M-3_ was predominantly carried by IncL/M plasmid and IncI1 [7]. In the present study, *K. pneumoniae* from in admission patients showed the presence of rare ST types, possibly reflecting the isolates in the community.

Among *E. coli* isolates, we observed 9 ST types of the most common ST131(30.9%), ST38(11.8%), ST95(10.3%), ST316 and ST405. Interestingly, all isolates from ST131 were of phylogroup B2 and all but one were of O25bST131 clone. Our findings showed that O25bST131 represented the major clone among fecal *E. coli* isolates harboring different ESBL genes—CTX-M-15, CTX-M-3, CTX-M-14, CTX-M-1, and SHV-12. Similar to results of other authors, we have observed it mainly among patients at admission, showing that the community could be a reservoir for this clone [35,36]. ST131 was more likely to be transmitted between members of the same family than within patients in the hospital environment [37]

The fact that O25bST131 members were detected also among medical personnel is alarming. Strict infection control measures and education of personnel were applied and six months later, ESBL-producing isolates were no more found among the medical staff (personal communication). O25bST131 is a widely distributed, highly virulent, and epidemic PanEuropean clone, that could easily be transmitted all over the world and is associated with CTX-M-15, OXA-1, and quinolone resistance. Our results showed the potential of this clone to produce a wide range of enzymes—in addition to CTX-M-15, also CTX-M-3, CTX-M -14, CTX-M-1, and SHV-12. Its virulence enables the bacteria to cause both hospital- and community acquired infections [38]. Some authors report an association of O25bST131 with prolonged gut carriage [39].

Our isolates from the ST95 (B2 phylogroup) clone were mainly associated with CTX-M-14 production, five of seven ST95 isolates produced CTX-M-14. This clone has been referred to as an important clinical clone associated with urinary and bloodstream infection [36,40,41]. ST38, belonged to D phylogroup, also has been commonly reported in clinical *E. coli* isolates and reported as a high-risk clone [4]. In this study, ST38 takes the second place after ST131 according to its frequency and was found to produce CTX-M-3, CTX-M-14 and CTX-M-15 enzymes.

In our study, two isolates of *E. coli* ST405 produced CTX-M-15. This clone was previously detected in Bulgaria as a carrier of NDM-1 carbapenemases [42]. *E coli* ST405 is an emerging urosepsis pathogen, reported to carry *bla* _CTX-M_, *bla* _NDM_, and a number of virulence genes comparable with O25b:H4-ST131 [43].

*E. coli* phylogroups B1 and A have been reported as commensal gut isolates and B2 and D to have pathogenic potential [4]. In the present study, the phylotyping showed that isolates members of B2 and D phylogroups were prevalent. ST 38, ST 95 and ST405 belonged to more virulent B2 and D groups and have been associated with increased virulence [4].

Among CTX-M-3 carriers, two types of plasmids were detected. IncL/M plasmid has been responsible for transmission among CTX-M-3-producing *K. pneumoniae, Enterobacter* spp. and *S. sonnei* isolates. In addition to IncL/M, among *E. coli* IncI1 has been confirmed too. CTX-M-15 production was associated with IncF and HI2 plasmids. The transconjugants showed resistance not only to cephalosporins third generation but also to aminoglycosides and co-trimoxazole. These antimicrobials could also make selective pressure. The high number of the isolated *E. coli* ST types showed that in addition to the plasmid, clonal transmission also plays an important role in distribution of ESBL genes. *Bla*KPC-2 were strongly associated with FIIAs replicon plasmids, as was reported previously [13].

In conclusion, this is the first report in Bulgaria about gut colonization with ESBL and/or carbapenemase producers. A high frequency of ESBL gut producers among hospitalized patients (42.4%) and in patients at admission (25.5%) was found. Three percent of isolates were KPC-2-producing *K. pneumoniae*. Only 4% of the medical staff evaluated carried ESBLs. The prevailing number of isolates were members of two highly epidemic and virulent PanEuropean clones, ST15 *K. pneumoniae* (40%, including two isolates from the medical staff) and ST131 clone (31%, also including 2 isolates from the medical staff). The isolates produced a wide range of ESBLs—CTX-M-15 (47%), CTX-M-3 (39%), and CTX-M-14 (11%).

In the present COVID pandemic situation, the antimicrobial overconsumption will increase the selective pressure both in the hospitals and in the community and may increase the rate and dissemination of ESBL/carbapenemase producers. Further studies should closely monitor this potential negative trend. Routine screening at hospital admission and during the hospital stay, as well as strict infection control measures should be widely implemented. In the present situation, adequate antimicrobial stewardship strategies are of critical importance for the restriction of unnecessary antimicrobial usage and prevention of the wide dissemination of problematic MDR bacteria.

## Figures and Tables

**Table 1:** Distribution of extended spectrum beta-lactamases (ESBLs)/carbapenemases according to species and patient’s type.

ESBL. Species	CTX-M-3 Number H/PA	CTX-M-15 Number H/PA	KPC-2 + CTX-M-15 Number H/PA	CTX-M-14 Number H/PA	CTX-M-1 Number H/PA	SHV-12 Number H/PA
*K. pneumoniae* * n* = 20	**4**/2	**11**/0	**3**/0			
*E. coli n* = 68	**9**/18	**6**/22		**0**/1	**8**/3	**0**/1
*E. cloacae* complex *n* = 5.	**1**/0	**2**/**1.**				
*K. aerogenes n* = 2	**2**/0					
*C. freundii n* = 1		**1**/0				
*S. sonnei n* = 2	**0**/2					
Total *n* = 98	**16**/22	**20**/23	**3**/0	**0**/1	**8**/3	**1**/1

Abbreviations: H—hospitalized patients, in bold, PA patients at admission.

**Table 2:** ESBL/carbapenemases, Multilocus Sequence Typing (MLST), replicon typing, and transconjugant characteristics of 20 *K. pneumoniae* isolates.

Patients, Type	ESBL	MLST Types	Phenotype of Transconjugants	Replicon Type
Hospitalized (14)	CTX-M-3 (4)	ST17(4)	CTX,TOB.GEN.AMI,SXT(2)	L/M(2)
	CTX-M-15 (7)	ST15(5) *	-	
		ST340(1) ST16(1)	-	
	KPC-2+CTX-M-15 (3)	ST15(3)	IMP,MER,PIP/TAZ (2)	FIA,FIIAs (2)
At admission (6)	CTX-M-3 (2)	ST37(2)	CTX, GEN (2)	L/M(2)
	CTX-M-15 (4)	ST215(1) ST359(1) ST873(2)	CTX,CAZ,TOB,GEN,TET CTX,CAZ,TOB,GEN,CIP -	F (1) NT (1)

Abbreviations: CTX, cefotaxime; CAZ, ceftazidime; TOB, tobramycin, GEN, gentamycin; AMI, amikacin; TET, tetracycline, SXT, trimethoprim/sulfamethoxazole; * including two isolates from the medical personnel.

**Table 3:** ESBLs, MLST, replicon typing and transconjugant characteristics of 68 *E*. *coli* isolates.

	ESBL	ERIC	MLST	Phylogroup	Phenotype of Transconjugants	Replicon Type
Isolates from HP *n* = 23	CTX-M-3 *n* = 9	f(5)	ST131 *	A	CTX (2)	I1(2)
		b(1)	ST 38	D	CTX (1)	I1(1)
		uni(3)	ND	A	CTX,TOB,GEN,AMI.SXT(1) CTX, SXT (1)	L/M(1) I1(1)
	CTX-M-14 *n* = 8	c(2)	ST 43	A	-	
		a(4)	ST 95	B2	CTX(2)	F(2)
		b(2)	ST 38	D	-	
	CTX-M-15 *n* = 6	f(3)	ST131	B2	CTX,CAZ,TOB,GEN,TET(2)	FIA,F(2)
		d(2)	ST405	D	CTX,CAZ,TOB,TET,SXT(1)	FIA,F(1)
		l(1)	ST316	A	-	
Isoaltes from PA *n* = 45	SHV-12 *n* = 1	f(1)	ST131	B2	-	
	CTX-M-1 *n* = 1	f(1)	ST131	B2	-	
	CTX-M-3 *n* = 18	f(5) **	ST131	B2	CTX(2) CTX,TOB,GEN,AMI(1) CTX,TOB,GEN,SXT,CHL(1)	I1(2) L/M(1) FIIAs(1)
		l(3)	ST316	A	CTX(1)	I1(1)
		b(2)	ST 38	D	CTX(1)	I1(1)
		m(2)	ST676	B2	CTX,GEN(1)	L/M(1)
		a(1)	ST 95	B2	CTX(1)	I1(1)
		t(3)	ST 517	B1	CTX,GEN(1)	L/M(1)
		uni(2)	ND	A(2)	CTX(2)	I1(2)
	CTX-M-14 *n* = 3	a(1)	ST 95	B2	CTX(1)	F(1)
		f(1)	ST131	B2	CTX(1)	F(1)
		m(1)	ST676	B2		
	CTX-M-15 *n* = 22	f(5)	ST131	B2		
		l(3)	ST316	A	CTX,CAZ(2) CTX,CAZ,SXT(1)	F(3)
		a(1)	ST 95	B2		
		m(2)	ST676	B2	CTX,CAZ(1)	F(1)
		*n*(2)	ST540	A	CTX,CAZ(1)	NT(1)
		b(3)	ST38	D	CTX,CAZ(1)	NT(1)
		uni (6)	ND	A(5),B1(1)		

Abbreviations: CTX, cefotaxime; CAZ, ceftazidime; TOB, tobramycin, GEN, gentamycin; AMI, amikacin; TET, tetracycline, SXT, trimethoprim/sulfamethoxazole; ND—not determined, * including two isolates from the medical personnel; ** including one *pab* negative isolate; HP, hospitalized patients; PA, patients at admission.
